# Analysis of the Complete Plastomes of 31 Species of *Hoya* Group: Insights Into Their Comparative Genomics and Phylogenetic Relationships

**DOI:** 10.3389/fpls.2021.814833

**Published:** 2022-02-08

**Authors:** Wyclif Ochieng Odago, Emmanuel Nyongesa Waswa, Consolata Nanjala, Elizabeth Syowai Mutinda, Vincent Okelo Wanga, Elijah Mbandi Mkala, Millicent Akinyi Oulo, Yan Wang, Cai-Fei Zhang, Guang-Wan Hu, Qing-Feng Wang

**Affiliations:** ^1^CAS Key Laboratory of Plant Germplasm Enhancement and Specialty Agriculture, Wuhan Botanical Garden, Chinese Academy of Sciences, Wuhan, China; ^2^Sino-Africa Joint Research Center, Chinese Academy of Sciences, Wuhan, China; ^3^University of Chinese Academy of Sciences, Beijing, China

**Keywords:** chloroplast, *Hoya*, *Dischidia*, phylogeny, barcoding, genomics

## Abstract

*Hoya* is a genus in Apocynaceae-Asclepiadoideae, known for its showy wax flowers, making it a popular ornamental plant. However, phylogenetic relationships among most *Hoya* species are not yet fully resolved. In this study, we sequenced 31 plastomes of *Hoya* group species using genome skimming data and carried out multiple analyses to understand genome variation to resolve the phylogenetic positions of some newly sequenced Chinese endemic species. We also screened possible hotspots, *trnT-trnL-trnF, psba-trnH*, and *trnG-UCC*, *ndhF*, *ycf1, matK, rps16*, and *accD* genes that could be used as molecular markers for DNA barcoding and species identification. Using maximum likelihood (ML) and Bayesian Inference (BI), a species phylogeny was constructed. The newly assembled plastomes genomes showed the quasi-tripartite structure characteristic for *Hoya* and *Dischidia* with a reduced small single copy (SSC) and extremely enlarged inverted repeats (IR). The lengths ranged from 175,404 bp in *Hoya lacunosa* to 179,069 bp in *H. ariadna*. The large single copy (LSC) regions ranged from 80,795 bp (*Hoya liangii*) to 92,072 bp (*Hoya_*sp2*_*ZCF6006). The massively expanded IR regions were relatively conserved in length, with the small single-copy region reduced to a single gene, *ndhF*. We identified 235 long dispersed repeats (LDRs) and ten highly divergent hotspots in the 31 *Hoya* plastomes, which can be used as DNA barcodes for species identification. The phylogeny supports *Clemensiella* as a distinct genus. *Hoya ignorata* is resolved as a relative to Clade VI species. This study discloses the advantages of using Plastome genome data to study phylogenetic relationships.

## Introduction

*Hoya* is the second largest genus in Apocynaceae-Asclepiadoideae with at least 300 species, after *Ceropegia* L. (ca. 357 species) (Bruyns et al., 2017). The genus consists of sub-shrub lianas composed of epiphytic climbers that grow in dense tropical forests of South, Southeast and East Asia, and Australasia (Lamb et al., 2016). The flowers have rotated corollas, staminal coronas with revolute margins, pollinia with pellucid margins, and elongated, slender, and fusiform seeds with hairs attached at their terminal part (Omlor, 1998). Due to their showy flowers, ease of growing, and popularity as ornamental plants, they have been grown in botanical gardens around the world. However, overexploitation from the wild may exist.

*Dischidia* is a genus closely related to the genus *Hoya* found in South East Asia. It consists of approximately 80 species (Livshultz et al., 2005). The best-known species is *Dischidia major*, which has pitcher leaves used as nesting sites by arboreal ants. Unlike *Hoya*, *Dischidia* is poorly known and has not been studied closely. Some species have oil-rich structures in the seeds that are attractive to ants and may facilitate seed dispersal (Rintz, 1980).

The first broadly sampled *Hoya* phylogeny used a few chloroplast loci (*matK* gene; *trnH-psbA, psbA*-*atpB, trnT*-*trnL*, and *trnL*-*trnF* intergenic spacers) and have been heavily informed by nuclear (ITS and 5′ ETS) (Wanntorp et al., 2006, 2014). They erected six main intrageneric lineages (Clade I–VI) comprising many species with mostly congruent plastid and nuclear affinities. Most interclade relationships remained ambiguous even in the study by Rodda et al. (2020a) with more samples. Using first phylogenomic data from complete plastomes (Rodda and Niissalo, 2021) further resolved the interclade and some intra-clade relationships and re-defined *Hoya* s.str. as comprising only Clade III–VI (Rodda and Niissalo, 2021). Phylogenetic analyses of 42 *Hoya* plastomes using exons larger than 90 bp erected a new clade Y, which was not captured by Rodda et al. (2020b) demonstrating that any new data may return further insights.

Nevertheless, all the taxonomic and phylogenetic conclusions are inferred from unreliable and dynamic morphological features or DNA fragments with limited polymorphic information loci, which may inevitably bias the phylogenetic reference (Philippe et al., 2011). Additionally, future studies on *Hoya* will pay more attention to population genetics and the true biogeographic origin. All these studies rely on high-resolution molecular markers and robust phylogeny, but the limited and low-resolution DNA markers heavily inhibited the comprehensive evaluation of *Hoya* resources. Therefore, it is imperative to develop efficient molecular markers to resolve the current problems.

The plastome, in addition to the nuclear and mitochondrial genomes, is one of the genetic systems that help to understand genetically inherited traits as it exhibits multiple evolutionary histories in angiosperms. Generally, phylogenetic inferences using nuclear genomes are unrealistic for their costly situation and lack enough genomic data (Wang et al., 2014; Olsen et al., 2016). On the other hand, mitochondrial genomes are unsuitable for phylogenetic analysis due to their slow evolutionary rate (Palmer and Herbon, 1988). Plastomes have independent evolutionary routes and are characterized by uniparental inheritance, moderate nucleotide substitutions, haploid status, and no homologous recombination compared to mitochondrion genomes (Shaw et al., 2005; Hansen et al., 2007). In parallel to that, these features of plastomes make them particularly suitable for phylogenetic and biogeographic studies of plants (Huang et al., 2014; Walker et al., 2014; Attigala et al., 2016). With the pileup of angiosperm plastomes, comparative genomics and phylogenomics of closely related plastomes are very useful for grasping the genome evolution regarding structure variations, nucleotide substitutions, and gene losses (Barrett et al., 2016; Raman and Park, 2016; Hu et al., 2017).

The first sequenced complete plastomes of *Hoya* were as follows: *Hoya carnosa* (*H. carnosa*) reported by Wei et al. (2020), *Hoya liangii* (*H. liangii*) and *Hoya pottsii* (*H. pottsii*) reported by Tan et al. (2018). Additionally, Rodda and Niissalo (2021) reported 20 newly sequenced plastomes of species in the *Hoya* group. While plastomes usually contain ≈110–130 protein-coding genes, ≈30 transfer RNAs (tRNAs) genes, and ≈4 ribosomal RNAs (rRNA) (Guisinger et al., 2010) organized in the LSC and small single copy (SSC), separated by the IR regions (including all rRNA gene), the *Hoya* plastomes have lost almost the entire SSC region due to the expansion of the two inverted repeats (IRs) (Wei et al., 2020; Rodda and Niissalo, 2021). Currently, the cp genomes of *Hoya* are rare and far much less for the clades *Clemensiella* and *Eriostemma*.

We sequenced complete plastomes of 31 species of the *Hoya* group (*Dischidia australis, D. griffithii, D. nummularia, D. ruscifolia, Hoya angustifolia* (*pottsii*), *H. ariadna, H. caudata, H. chinghungensis, H. commutata, H. dimorpha, H. griffithii, H. kerrii, H. lacunosa, H. lanceolata subs. Bella, H. liangii, H. longifolia, H. meliflua subs. fraterna, H. ovalifolia, H. pandurata, H. pottsii, H. pubicalyx, H. radicalis, H. rigida, H. sylvatica, Hoya* sp. 11 ZCF6107, *Hoya* sp. 2 ZCF6006, *Hoya* sp. 3 ZCF6076, *Hoya* sp. 4 ZCF6004, *Hoya* sp. 8 ZCF6076, *H. thomsonii*, and *H. volubilis*) then, conducted comparative genomics and phylogenomics analyses by integrating previously published cp genomes from Tan et al. (2018); Wei et al. (2020), and Rodda and Niissalo (2021). We aim to compare and characterize the cp genomes among selected species of *Hoya*, identify and select molecular markers suitable for population genetics, reconstruct the species relationships of the six extant clades of the *Hoya* group. This study provides useful genomic information for molecular evolutionary and phylogenetic studies of the *Hoya* group and genetic resources for breeding and improving the species.

## Materials and Methods

### Sampling, DNA Extraction, and Sequencing

A total of 31 *Hoya* group species (Supplementary Table 1) were collected from the orchards from Xishuangbanna Tropical Garden and South China Botanical Garden, CAS, and their voucher specimens were deposited at the herbarium of Wuhan Botanical Garden, CAS (HIB). Total genomic DNA was extracted from silica-dried leaves using a modified cetyl trimethylammonium bromide (CTAB) protocol (Li J. et al., 2013), and quality was assessed by agarose gel electrophoresis. Total DNA was sent to Novogene Company (Beijing, China)^1^ for short insert (350 bp) library construction and next-generation sequencing. Pair end reads of 2 × 150 bp for all tested species were generated on an Illumina Hiseq 4,000 genome analyzer platform. Original reads were filtered using the FASTX-Toolkit^2^ to acquire high-quality data by deleting adaptors and low-quality reads.

### Chloroplast Genome Assembly, Annotation, and Comparison

Filtered high-quality reads were assembled into complete plastomes using GetOrganelle v1.7.5 (Jin et al., 2020) with the following settings: word size set to (w -6), number of rounds to 10 (R -10). Finally, the complete paths were viewed in Bandage 0.8.1 (Wick et al., 2015).

The resulting complete circular plastomes were annotated by Plastid Genome Annotator (PGA) (Qu et al., 2019) and GeSeq (Tillich et al., 2017). The resulting sequences were manually checked in Geneious 8.0.4 (Kearse et al., 2012) using reference plastid genome *H. carnosa* (Wei et al., 2020) to avoid annotation errors. In addition, tRNAs were further verified using the tRNAScan-SE search server (Schattner et al., 2005). All newly assembled chloroplast genomes were deposited in GenBank (accession numbers are shown in Supplementary File 1). The circular genome map with structural features was generated using OGDRAW (Greiner et al., 2019).

### Genome Comparison

Out of 31 sequenced plastomes, 20 species were chosen to represent each clade for genome comparison using *H. carnosa* as the reference sequence. The plastomes were grouped into nine representatives for each clade except *Dischidia* clade then aligned in progressive mauve (Darling et al., 2010) implemented in mauve v.2.4.0 (Darling et al., 2004) to detect any form of rearrangement in *Hoya*. The IR expansion and contraction among 20 chloroplast genomes were visualized by the online program IRscope (Amiryousefi et al., 2018).

### Characterization of Repetitive Sequences

Simple sequence repeats (SSRs) across the 31 plastomes were extracted using the online web tool MISA^3^ (Beier et al., 2017) with the following parameters: ten repetitions for mononucleotide motifs, eight for dinucleotide motifs, and three for Penta and hexanucleotide motifs. Identification of the long dispersed repeats (LDRs): forward (F), palindromic (P), reverse (R), and complement (C) repeats analysis was done using the REPuter program^4^, with a minimum repeat size of 30 bp and a Hamming distance of 3 (Kurtz et al., 2001). Nucleotide diversity (*Pi*) was calculated by sliding window analysis conducted in DnaSP v.6.11.01 (Librado and Rozas, 2009), using a window length of 600 bp and a step size of 200 bp.

### Phylogenetic Analysis

The phylogenetic tree was constructed based on 72 protein-coding genes of 45,624 characters common among all the 55 *Hoya* group species and the two outgroup species; *Jasminanthes maingayi* and *Marsdenia flavescens* (Supplementary File 1). The nucleotide sequences were aligned using MAFFT v.7.2.2 (Katoh and Standley, 2013). Each alignment sequence was first trimmed using TrimAI v.1.2 (Capella-Gutiérrez et al., 2009) with default settings to reduce poorly aligned regions. The resulting trimmed alignments were then filtered with Gblocks (Talavera and Castresana, 2007) to clean the sequences from poorly aligned positions and too divergent regions. The final alignment for all datasets was concatenated in Phylo Suite v.1.2.1 (Zhang et al., 2020). Using Bayesian Information Criterion (BIC), the best-fit models for the phylogenetic analysis were GTR, GTR + G, and GTR + I + G, under settings (R cluster) for the concatenated alignment as implemented in ModelFinder. This study employed two different phylogenetic algorithms/optimality criteria: maximum likelihood (ML) and Bayesian Inference (BI). The ML tree was constructed using IQ-tree (Nguyen et al., 2015) implemented in Phylosuite with the best-fit models determined by ModelFinder and 1,000 replicates for ultrafast bootstrapping (Minh et al., 2013). BIs were performed by MrBayes v.3.2.7 (Ronquist et al., 2012) under the GTR + G model with four chains and two parallel runs. The Monte Carlo Markov chains (MCMCs) were run for 10 million generations and sampled at a frequency of every 1,000 generations. The first 25% of the trees were discarded as burn-in, and the remaining trees were used to build a majority-rule consensus tree and establish posterior probability values for each branch. The stationarity was considered to be met since the average SD of split frequencies remained below 0.115631. The final phylogenetic results were visualized with FigTree v.1.4.4 (Rambaut, 2018).

### Detection of Selection Pressure

We applied the site model method implemented in CodeML (Gao et al., 2019) to detect positively selected sites in *Hoya* group species. Our selection analysis was based on 72 protein coding-region sequences after all stop codons were removed. The positive selection models (M2a and M8) and their respective null models (M1a and M7) implemented in the site model were used to conduct the adaptive evolution analysis. Likelihood ratio tests (LRTs) were performed two times to compare the difference in the log-likelihoods between the nested codon-based models (Yang, 1998). The Bayes Empirical Bayes (BEB) method was used to identify the most likely codons under positive selection (Yang et al., 2005).

## Results

### Characterization of the Chloroplast Genomes

**FIGURE 1:**
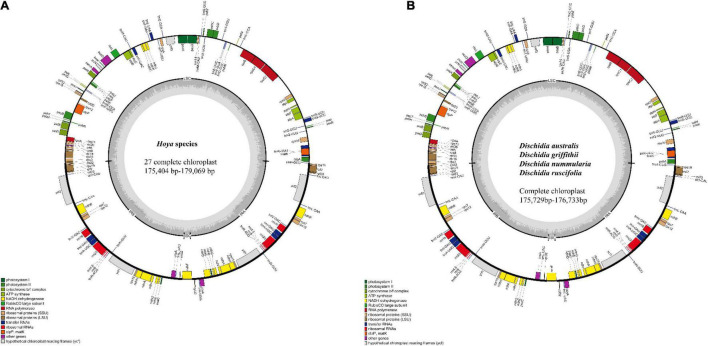
The genome maps of **(A)** 27 *Hoya* species; **(B)** 4 *Dischidia* species. The genes inside the circle are transcribed in the clockwise direction and those outside in the anticlockwise direction. The different colors represent the genes of different functional groups. The thick lines denote the extent of IRa and IRb, which separates the chloroplast genome into LSC and SSC. LSC: large single copy; SSC: small single copy. Plastome structure variation.

Approximately 173.84 GB of paired-end quality reads were obtained from Illumina sequencing for the 31 *Hoya* group species from China (Table 1). All plastomes showed the quasi-tripartite structure characteristic for *Dischidia* and *Hoya* with a strongly reduced SSC and significantly enlarged IR regions (Figures 1A,B). Their lengths varied in sizes ranging from 175,404 bp in *Hoya lacunosa* to 179,069 bp in *H. ariadna*, mainly because of length variation in LSC (80,795 bp in *H. liangii* to 92,072 bp in *Hoya* sp2 ZCF6006). The SSC region only includes a single gene (*ndhF*), ranging from 2,265 in *Hoya pandurata* to 2,306 in *Hoya caudata.* Slight variation characterizes the sizes of their IR regions and overall guanine-cytosine (GC) contents (Table 1).

**TABLE 1:** Summary of 31 *Hoya* plastome features that were assembled and annotated.

Species name	Total length (bp)	LSC (bp)	SSC (bp)	IR (bp)	Total GC content (%)	Total no. of tRNA	Total no. of rRNA	Total no. of genes
*Dischidia australis*	176,733	91,267	2,298	41,584	37.1	38 (8)	8 (4)	143 (113)
*Dischidia griffithii*	176,733	91,267	2,298	41,584	37.0	38 (8)	8 (4)	143 (113)
*Dischidia nummularia*	175,529	90,407	2,292	41,415	38.6	38 (8)	8 (4)	143 (114)
*Dischidia ruscifolia*	175,887	90,945	2,298	41,322	37.1	38 (8)	8 (4)	143 (113)
*Hoya angustifolia*	175,940	90,482	2,294	41,582	38.8	38 (8)	8 (4)	142 (113)
*Hoya ariadna*	179,056	92,383	2,293	42,190	38.6	38 (8)	8 (4)	142 (114)
*Hoya caudate*	176,887	91,553	2,306	41,514	38.6	38 (8)	8 (4)	142 (114)
*Hoya chinghungensis*	176,221	90,614	2,297	41,655	38.7	38 (8)	8 (4)	142 (114)
*Hoya commutate*	176,458	91,191	2,293	41,487	38.7	38 (8)	8 (4)	142 (114)
*Hoya dimorpha*	177,575	91,824	2,303	41,724	37.1	38 (8)	8 (4)	143 (113)
*Hoya griffithii*	177,132	91,278	2,288	41,783	38.7	38 (8)	8 (4)	143 (113)
*Hoya kerrii*	176,967	91,514	2,285	41,584	38.6	38 (8)	8 (4)	142 (114)
*Hoya lacunose*	175,404	90,038	2,294	41,536	38.6	38 (8)	8 (4)	142 (114)
*Hoya lanceolata subs. bella*	176,739	90,927	2,304	41,754	38.6	38 (8)	8 (4)	142 (114)
*Hoya liangii*	177,005	91,249	2,288	41,734	36.9	38 (8)	8 (4)	143 (113)
*Hoya longifolia*	177,990	91,318	2,288	41,692	37.1	38 (8)	8 (4)	142 (114)
*Hoya meliflua subs. fraterna*	177,130	91,658	2,302	41,585	36.9	38 (8)	8 (4)	142 (114)
*Hoya ovalifolia*	176,757	91,330	2,297	41,565	38.6	38 (8)	8 (4)	142 (114)
*Hoya pandurate*	176,320	90,889	2,265	41,583	38.6	38 (8)	8 (4)	142 (114)
*Hoya pottsii*	175,818	90,468	2,294	41,528	38.6	38 (8)	8 (4)	142 (114)
*Hoya pubicalyx*	176,829	91,376	2,285	41,584	36.9	38 (8)	8 (4)	142 (114)
*Hoya radicalis*	177,347	91,218	2,293	41,918	37	38 (8)	8 (4)	142 (114)
*Hoya rigida*	176,474	91,026	2,288	41,580	37	38 (8)	8 (4)	143 (113)
*Hoya sylvatica*	175,950	90,468	2,294	41,594	37	38 (8)	8 (4)	142 (114)
*Hoya* sp. 11 ZCF6107	175,905	90,489	2,294	41,561	37	38 (8)	8 (4)	142 (114)
*Hoya* sp. 2 ZCF6006	178,241	92,072	2,305	41,932	37	38 (8)	8 (4)	143 (113)
*Hoya* sp. 3 *ZCF6076*	177,781	91,192	2,297	42,146	37.1	38 (8)	8 (4)	142 (114)
*Hoya* sp. 4 ZCF6004	178,838	92,175	2,295	42,184	37	38 (8)	8 (4)	143 (113)
*Hoya* sp. 8 ZCF6076	177,198	91,618	2,294	41,643	37	38 (8)	8 (4)	143 (113)
*Hoya thomsonii*	161,420	80,795	2,289	39,168	37	38 (8)	8 (4)	143 (113)
*Hoya volubilis*	176,525	91,749	2,294	41,241	37.1	38 (8)	8 (4)	142 (113)

*The standard gene number in Hoya is 143, 113–114 genes were unique, and 17 genes were duplicated in the two IRs (Table 1). ycf15 gene is missing in three of the Dischidia species and ten species of Hoya. All chloroplast genomes include 79–80 protein-coding genes, 30 tRNA genes, and four rRNAs; the IR regions comprise 29 genes (17 protein-coding, 8 tRNA, and 4 rRNA genes).*

### Plastome Structure Variation

**FIGURE 2:**
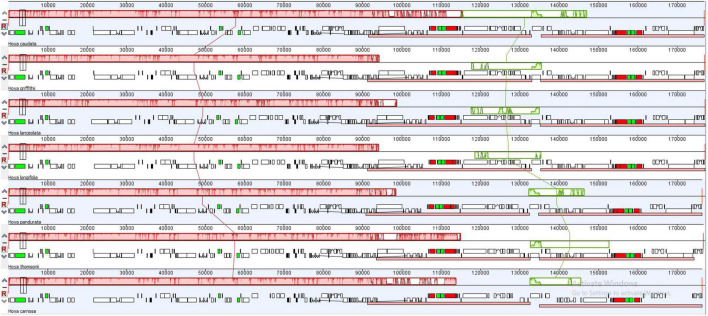
Rearrangements in 7 *Hoya* plastomes using the mauve multiple alignment algorithm. Different colors represent different collinear blocks. The lines linking the collinear blocks represent homology between different genomes. The scale above each genome indicates nucleotide positions, and the white regions represent elements specific to a genome. IR contraction and expansion.

All the *Hoya* plastomes showed the same order and orientation of syntenic blocks (Figure 2), indicating that *Hoya* plastomes are highly conserved and collinear. Nevertheless, a few local changes representing variable regions were detected, with several evident inversions mainly located in SC regions, especially within the nucleotide of 125,000–145,000 bp.

### Inverted Repeats Contraction and Expansion

The LSC/IRb boundary was consistently located downstream of the *rpl22* gene within the 3′ part of the *rpl22* gene (Figure 6). The IRb/SSC SSC/IRa junctions were located 35–40 bp upstream and downstream of the *ndhF* gene (Figure 6). The IRa/LSC junction fell within the *rps19-trnH* (GUG) spacer. IR contraction and expansion in the *Hoya* plastomes ultimately lead to the length variations of the four structural segments and whole-genome sequences.

**FIGURE 3:**
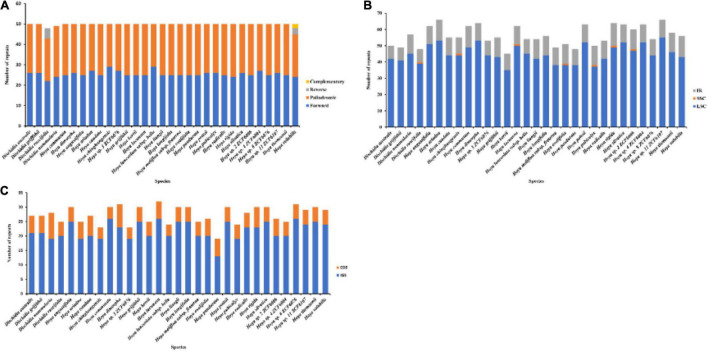
Analyses of repeat sequences and SSRs in 31 *Hoya* group plastomes. **(A)** Frequency of the four repeat types. **(B)** Frequency of SSRs in LSC, SSC, and IR. **(C)** Frequency of SSRs in IGS and CDS. LSC, large single copy; SSC, small single copy; SSR, simple sequence repeats.

**FIGURE 4:**
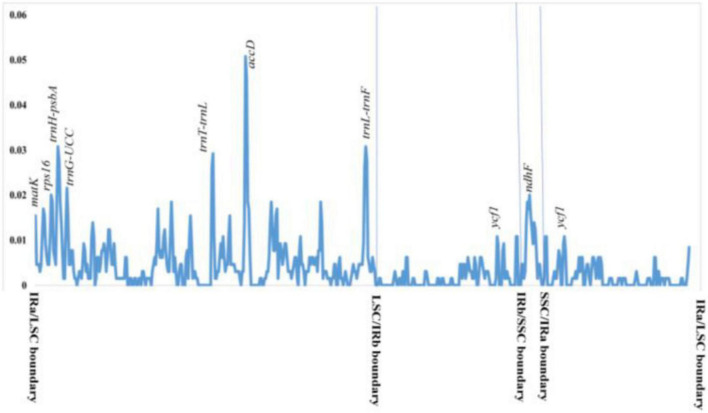
Nucleotide diversity (Pi) in 31 *Hoya* group complete plastomes. The values represent different diversity for different genes and regions.

**FIGURE 5:**
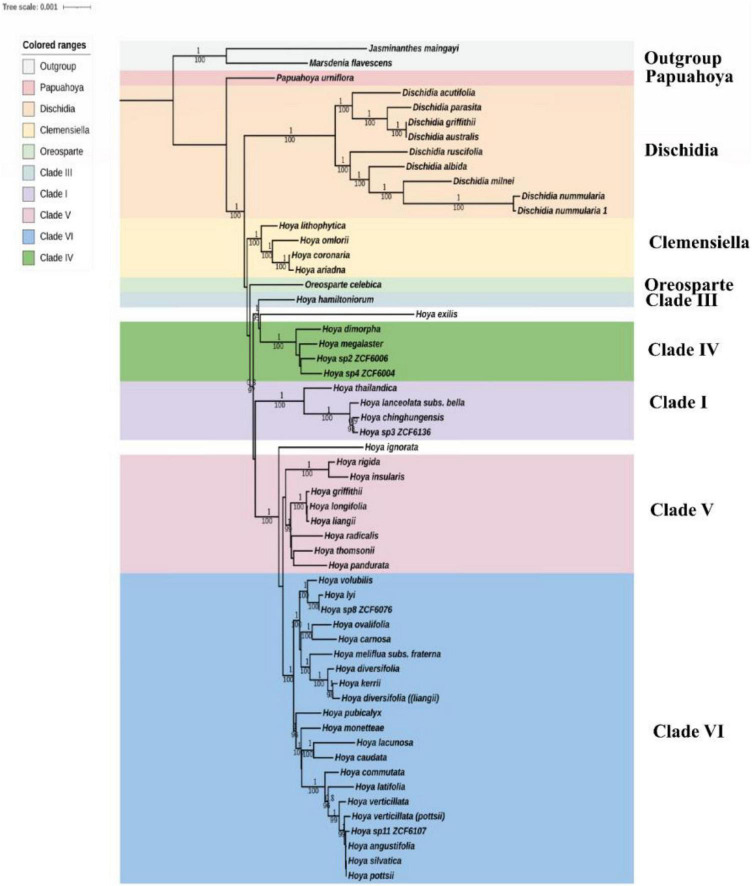
Phylogenetic tree of *Hoya* inferred from 57 species using ML. CDS tree reconstruction is rooted with *Marsdenia* and *Jasminanthes* as outgroups. Bootstrap support values and Bayesian inferred posterior probabilities are given below and above the branches, respectively. Different colors represent different clades following Wanntorp et al. (2014) and Rodda and Niissalo (2021).

**FIGURE 6:**
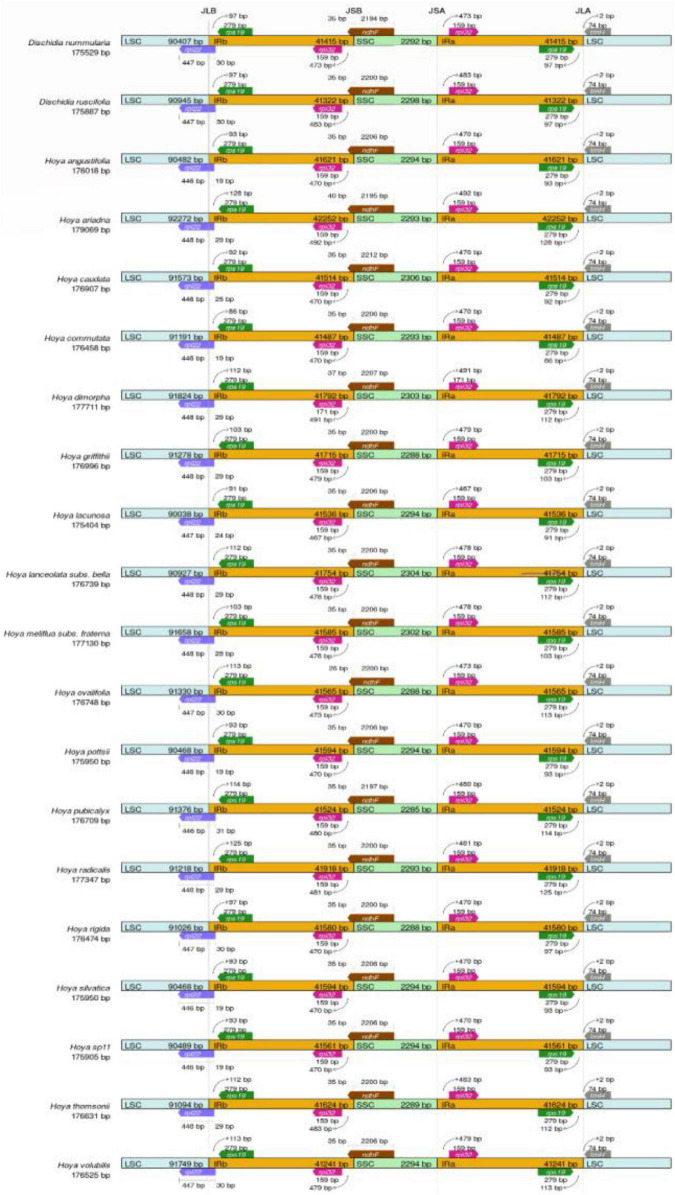
Junction sites comparison of LSC, SSC, and IR for 20 *Hoya* group plastomes. JLB, junction line between LSC and IRb; JSB, junction line between IRb and SSC; JSA, junction line between SSC and IRa; JLA, junction line between IRa and LSC; LSC, large single copy; SSC, small single copy.

### Repeat Analysis

We identified a total number of 235 SSR motifs in our set of 31 *Hoya* group plastomes (four *Dischidia*, 27 *Hoya*). Mono-nucleotide repeats were more prevalent and most of them belonged to the A/T type (163 repeats) followed by AAG/CTT (60 repeats). Penta and hexanucleotides were rare (5 repeats each); the rarest were A/G (2 repeats). Additionally, four types of LDRs (forward, palindromic, reverse, and complement), each with a motif length longer than 30 bp, were detected (Figure 3 and Supplementary Table 1). Finally, a total of 1,446 repeats, 709 forward (F) and 725 palindromic (P), were detected across all chloroplast genomes with eight reverse (R) and four complementary (C) in the plastomes of *Dischidia ruscifolia* and *Hoya volubilis*, respectively.

Nucleotide diversity (*Pi*) was calculated by sliding window analysis to observe sequence divergence and determine highly divergent hotspots. Both single-copy regions were identified as having greater sequence divergence than the IR region (Figure 4). With a Pi-value cut-off point of 0.02, eight highly variable gene regions were identified: intergenic spacers (*trnT-trnL-trnF, psba-trnH*, and *trnG-UCC*), *ndhF*, *ycf1, matK, rps16*, and *accD* genes. Six of the highly variable regions were located in the LSC, while one was in the SSC and IR regions.

### The Synonymous and Non-synonymous Substitution Rate Analysis

Using *Jasminanthes maingayi* as the outgroup, we computed and compared the dN/dS of *H. ariadna*, *H. caudata, H. chinghungensis, H. pubicalyx, H. longifolia, H. thomsonii*, and *H. rigida*. According to the statistical neutrality test, 10 genes in the seven plastomes of selected *Hoya* species were under positive selection. The genes were majorly involved in adenosine triphosphate (ATP) synthesis (*atpA, atpB, atpE*, and *petL*), RNA processing (*matK*), NADH dehydrogenase (*ndhA, ndhB*, and *ndhD*), and other genes (*accD* and *clpP*) (Table 2). According to the M8 model, *atpA* harbored four sites under positive selection, followed by *atpB*, which had three sites. However, the LRT indicated that the models (M2a and M8) were significantly better than the control models (M1a and M7), proving the presence of codons under positive selection. Further analysis from BEB scores indicated an intense positive selection pressure on 15 codons (Table 2).

**TABLE 2:** Positive selected sites detected in the cp genomes of Hoya.

Gene names	M8		M2a	
	Selected sites	Pr (w > 1)	Selected sites	Pr (w > 1)
*AccD*	446L	0.992**	446L	0.983*
*AtpA*	2693Q	0.998**	2693Q	0.994*
	2694C	0.970*	2694C	0.93
	2695A	0.970*	2695A	0.928
	2741N	0.964*	2741N	0.913
*AtpB*	4499V	0.969*	4499V	0.928
	5071F	0.978*	5071F	0.952*
	5182I	1.000**	5182I	0.999**
*AtpE*	5693I	0.976*	5693I	0.948
*ClpP*	9580E	0.971*	9580E	0.931
*MatK*	11800I	0.968*	11800I	0.923
*NdhA*	12303L	0.998**	12303L	0.994**
	12375W	0.994**	12375W	0.988*
*NdhB*	13014S	0.967*	13014S	
*NdhD*	15799L	0.967*	15799L	0.921
*PetL*	17548F	0.966*	17548F	0.919

*p < 0.05; **p < 0.01.

### Phylogenetic Analysis

The phylogenetic tree was constructed based on 72 protein-coding genes common to all currently available *Hoya* group species (Supplementary File 1), i.e., two more representatives of Marsdenieae as outgroups to root the trees (Figure 5). The multiple sequence alignment comprised of protein-coding sequences with 45,624 characters and 2,332 variable sites. To compare the clades, we referred to Wanntorp et al. (2014), Rodda et al. (2020a), and Rodda and Niissalo (2021). The 55 in-group taxa formed nine distinct clades (Supplementary Figure 1), and both ML and BI yielded similar topologies (Figure 5). *Dischidia* is the second early diverged clade after *Papuahoya*, and it comprises nine species. *Dischidia griffithii* and *D. australis* form a clade with *D. parasita*, a species native to the Philippines. *Hoya ariadna* (a species belonging to the section *Eriostemma*) formed a stable sister relationship with species from clade II/*Clemensiella* [100% (Bootstrap support), 1 (Bayesian posterior probability)]. Within clade IV, there are two unidentified species (*Hoya* sp. 2 ZCF6006 and *Hoya* sp. 4 ZCF6004) that form a well-supported relationship to *Hoya megalaster* from section *Physostelma* (100% BS, 1 BPP). Clade V comprises eight montane subtropical species whose relationships are well supported (99% BS, 1 BPP). Clade VI is the most recently diverged and widespread in *Hoya* sensu lato. Furthermore, unidentified species (*Hoya* sp. 8 ZCF6076 and *Hoya* sp. 11 ZCF6107) are strongly supported to belong to clade VI (99–100 BS, 0.8–1 BPP), a lineage morphologically characterized by flowers with dark-colored nectar. *Hoya* sp. 3 ZCF6076 from Yunnan, China and *H. chinghungensis* are part of Clade I (BS = 100, PP = 1) and are closely related to *H. lanceolata* subsp. *bella*. Overall, the emended taxon set recovers the same intrageneric, interclade relationships as found by Rodda and Niissalo (2021).

## Discussion

### Chloroplast Genome Variation

The overall plastome sequences in the 31 *Hoya* group examined were highly conserved, and they did not exhibit the standard quadripartite structure similar to the other angiosperm plastomes. Their SSC was massively reduced with only one gene (*ndhF*) present and their IR regions much more prominent than other higher plants. This could be due to the complexity arising in assembling *Hoya* group plastomes, as reported by Crook (2017). Furthermore, the *ndhF* gene has been reported to be notoriously difficult to assemble, as they move between the IRb and SSC, sometimes straddling both regions (Davis and Soreng, 2010). Consequently, our data were manually adjusted using Geneious predictions to correct the poorly assembled plastomes. The exact number and contents of the genes were predicted in this study, suggesting that the evolution of the gene sequences was consistent across the 31 species. As a result, *Hoya* group species’ plastomes contain a total of 113–114 unique genes, such as 79–80 protein-coding genes, 30 tRNAs, and four rRNAs. The expansion and contraction of the IR is the main reason for variation in genomic size; rearrangements, such as inversion of genes and SSC, are common in plastome genomes (Liu et al., 2018). Additionally, it has been reported to have occurred in Marsdenieae and other Apocynaceae plastomes (Straub et al., 2011). Comparably, the newly sequenced *Hoya* (Figure 2) had similar rearrangements.

### Plastome Structure Variation

The sequence divergence of IR regions was lower compared to LSC and SSC, with the *accD* gene being the most divergent (Figure 4). This was caused by IR having very few protein-coding genes, short IGS, and mostly tRNAs and rRNA, which are more conserved than exons and introns of the protein-coding genes. Similarly, Straub et al. (2011) reported this on *Asclepias syriaca* but Rodda and Niissalo (2021) did not mention it in the recent *Hoya* plastomes. Intergenic regions, especially the *rpl32-trnL*, have been used for phylogenetic and evolutionary studies at the species level (Dong et al., 2012; Zecca et al., 2012; Jara-Arancio et al., 2018) due to its high nucleotide diversity, making it a mutationally active region. On the contrary, we discovered that the mutationally active plastome region in *Hoya* is the 3′ region of *ndhF* locus, *accD*, and *matK* genes, and the intergenic spacers (*trnT-trnL*, *trnH-psbA*, and *ycf1*) are the lowest (Figure 4). Similar to most land plants, the *ycf1* is the largest open reading frame (ORFs). It is located at the boundary of the IR and SSC, its diversity and length make it a better candidate for phylogenetic studies than other genes (Neubig et al., 2009). While still being one of the most variable regions, in the *Hoya* group, the *ycf1* is outcompeted by the only remaining SSC gene, the *ndhF* gene.

### Simple Sequence Repeats

Both LDRs and SSRs are useful genetic markers due to their abundance in chloroplast genomes, high degree of polymorphism, and co-dominance (Ivanovych and Volkov, 2018). In a previous study, Straub et al. (2011) focused only on the repeat sequences based on the nuclear genome. Similar to most angiosperms, sequence repeats for A/T were more prevalent than those of G/C in the *Hoya* group plastomes. This may represent bias in the base composition, which is potentially affected by the tendency of the genome to change to A-T rather than to G-C (Li X. et al., 2013). Notably, these microsatellites are likely to have originated from multiple paralogous loci of the *Hoya* group plastomes, as is the case with all the microsatellites. This is the ultimate proof of the utility of these markers for identifying intraspecific variation. There was variation in the distribution of LDRs and SSRs in non-coding intergeneric spacers (IGS) vs. coding region (CDS), with repeats being concentrated in IGS, in line with previous studies (e.g., Shen et al., 2018; Meng et al., 2019)

### Positive Selection

Testing synonymous and non-synonymous nucleotide substitution is vital in gene evolution studies (Drouin et al., 2008). Accordingly, the ratio ω = dN/dS has become a standard measure of selective pressure. Our study is the first to report on the selection pressures acting on protein-coding genes of *Hoya* s.l. From our findings, instances of multiple positive selections in different genes are involved in various functions, such as ATP synthesis (*atpA, atpB, atpE*, and *petL*), RNA processing (*matK*), NADH dehydrogenase (*ndhA, ndhB*, and *ndhD*), and other gene functions (*accD* and *clpP*). A total of 15 codons were detected to be under positive selection with high confidence levels (posterior probability > 0.95; Table 2).

### Phylogeny

Our plastome matrix of 72 protein-coding genes and 57 species based on ML and Bayesian analyses (Figure 5) represented the most extensive sampling of the protein-coding genes to date and was mainly in congruence with the previous results (Wanntorp et al., 2006, 2014; Rodda et al., 2020a; Rodda and Niissalo, 2021). The seven major clades established by Wanntorp et al. (2014) are well supported in our study (BS = 98–100, PP = 1.00). This reveals strong support for the relationships of these enigmatic species (*H. griffithii, H. Kerrii H. meliflua subs. fraterna, H. ovalifolia*, and *H. thomsonii*) sampled for the first time. These species were ambiguous taxa in the previous study done by Wanntorp et al. (2014) and their relationships could not be resolved using plastid loci *trnT-trnL*, *trnH-psbA*, and nuclear datasets (ITS and ITS). Moreover, Rodda and Niissalo (2021) did not resolve the relationships possibly due to sampling problems. Our analysis further confirms previous studies (Rodda et al., 2020a; Rodda and Niissalo, 2021), which recognized the monophyletic genera *Dischidia* and *Oreosparte* (BS = 100; PP = 1.0), and placed Clade II (*Clemensiella*) outside *Hoya* s.str. *Dischidia* remains the sister clade to *Hoya* s.l., i.e., *Oreosparte*. Phylogenetic relationships within the *Hoya* group on a large scale have been ambiguous mainly due to poor infrageneric resolution since there is no infrageneric system established up to date (Rodda et al., 2020b). For example, *Hoya caudata* is resolved as the sister of *H. lacunosa* (BS = 100, PP = 1.0), yet taxonomically and morphologically distinct. *Hoya caudata*, a widespread species distributed in S. Asia, Malesia, and Australasia, belongs to section *Peltostemma*, while *H. lacunosa* from S.W China, an oddball in Wanntorp et al. (2014) with conflicting nuclear and plastid affinities, is sect. *Otostemma*. Species of *Hoya* sect. *Otostemma* has revolute lobes, rotate corolla with boat-shaped corona segments. Their anthers are incumbent on the stigma, with the apex simple, acute, and pollinia attached at the base, close together, and linearly compressed (Kloppenburg, 2001). However, the *Hoya* sect. *Peltostemma* is distinguished through the inclined corona scales and long extended anther appendages. In addition, the stigma head is hollow on the point and slow to open. Clade II (sect. *Clemensiella*) on the other hand comprising *H. omlorii, H. coronaria, H. ariadna*, and *H. lithophytica* is sharply different from the rest of the *Hoya* group clades. Their branches are fleshy, their retinaculum is rather large, and the pollinia are more club shaped and moreover do not have a keel on the outer edge. Furthermore, Rodda and Niissalo (2021) suggested that it should be treated as a sub-genus of *Hoya*, despite its phylogenetic placement outside *Hoya* s.str. Our findings, such as the enigmatic species *Hoya ariadna*, confirm Rodda and Niissalo (2021) topology, hence, recognizing *Clemensiella* as a distinct genus, but the support of the critical branch remains ambiguous (BS = 76, PP = 0.7). In addition, the number of species belonging to *Clemensiella* is still small but with a wider search into the Malay Peninsula and the Sunda Islands, more species could be added. Species, such as *H. purpurea* Blume and *H. neoguineensis* Engler from New Guinea, *H. guppyi* Oliv., and *H. affinis* Hemsl. from the Solomon Islands, belong to this section/clade and they are particularly found in the forest edges and along streams (Kloppenburg, 2001; Wanntorp et al., 2014).

## Conclusion

Through the use of next-generation sequencing (NGS), 31 new plastomes of the *Hoya* group species were assembled and analyzed and used to complement existing data sets. The gene content, gene order, and GC contents were conserved in *Hoya* s.l. genomes, which share a unique chloroplast quasi-tripartite genome structure with *Dischidia*. Highly divergent regions (*ndhF, ycf1, rpl22, matK, trnT-trnL*, and *trnL-trnF*) and repeats (-mono, -tri, -Penta, and -hexanucleotides) that could potentially serve as molecular markers for phylogenetics were identified. All the phylogenetic analyses using the species of the *Hoya* group strongly supported the relationships among the species within the genus. The results and data presented in this study provide insights into the evolutionary relationships and biogeographic history of the *Hoya* group species. More detailed taxon sampling will further contribute to our understanding of phylogenetic dynamics in the *Hoya* group lineages.

## Data Availability Statement

The data presented in this study can be found in the GenBank repository. The accession number can be found in the Supplementary Material.

## Author Contributions

WO, C-FZ, G-WH, and Q-FW participated in the design of the study and carried out the experiments. YW, C-FZ, and G-WH collected the materials. WO, EW, CN, ESM, VW, EMM, and MO contributed to data analysis and draft manuscript writing. WO, C-FZ, G-WH, and VW revised the draft manuscript. All authors read and approved the final version of the manuscript.

## Conflict of Interest

The authors declare that the research was conducted in the absence of any commercial or financial relationships that could be construed as a potential conflict of interest.

## Publisher’s Note

All claims expressed in this article are solely those of the authors and do not necessarily represent those of their affiliated organizations, or those of the publisher, the editors and the reviewers. Any product that may be evaluated in this article, or claim that may be made by its manufacturer, is not guaranteed or endorsed by the publisher.

## Footnotes

1
http://www.novogene.com

2
http://hannonlab.cshl.edu/fastx_toolkit

3
http://pgrc.ipk-gatersleben.de/misa/

4
https://bibiserv.cebitec.uni-bielefeld.de/reputer
