# Supplementary figures and images for: Analysis of the Complete Plastomes of 31 Species of Hoya Group: Insights Into Their Comparative Genomics and Phylogenetic Relationships
Source: Front Plant Sci. 2022 Feb 8;12:814833. doi: 10.3389/fpls.2021.814833 (PMC8862764; doi:10.3389/fpls.2021.814833)

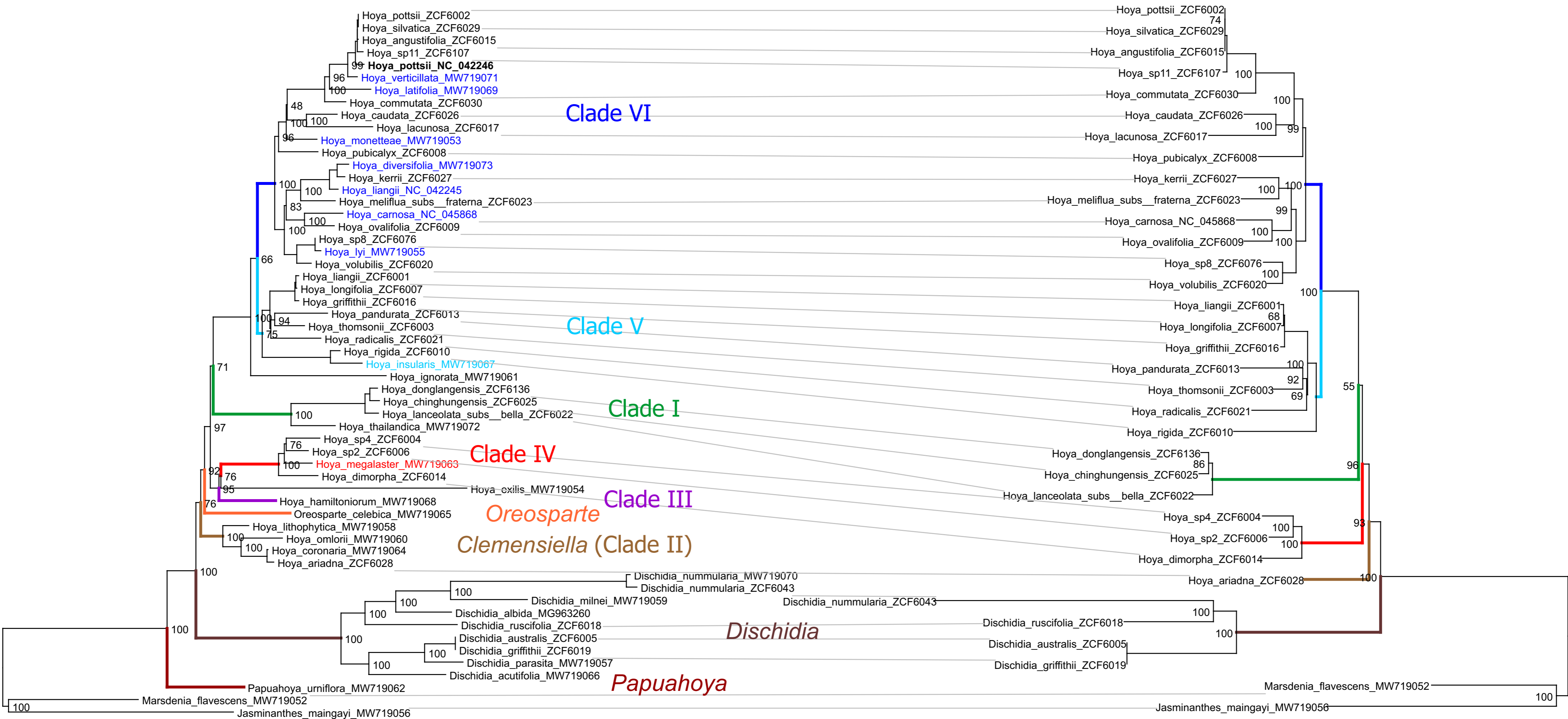

Figure S1: Hoya group clade sampling tanglegram for all the 31 species.

Supplement: Supplementary file 1 [file Data_Sheet_1.pdf]
