# Supplementary material for: Analysis of the Complete Plastomes of 31 Species of Hoya Group: Insights Into Their Comparative Genomics and Phylogenetic Relationships
Source: Front Plant Sci. 2022 Feb 8;12:814833. doi: 10.3389/fpls.2021.814833 (PMC8862764; doi:10.3389/fpls.2021.814833)
Supplement: Supplementary file 2 [file Table_1.docx]

**Supplementary table S1: Long dispersed repeats**

| Species | Forward | Palindromic | Reverse | Complementary |
| --- | --- | --- | --- | --- |
| *Dischidia australis* | 26 | 24 |  |  |
| *Dischidia griffithii* | 26 | 24 |  |  |
| *Dischidia ruscifolia* | 22 | 21 | 5 |  |
| *Dischidia nummularia* | 24 | 25 |  |  |
| *Hoya commutata* | 25 | 25 |  |  |
| *Hoya dimorpha* | 26 | 24 |  |  |
| *Hoya angustifolia* | 25 | 25 |  |  |
| *Hoya ariadna* | 27 | 23 |  |  |
| *Hoya caudata* | 25 | 25 |  |  |
| *Hoya chinghungensis* | 29 | 21 |  |  |
| *Hoya* sp. 3 ZCF6076 | 27 | 23 |  |  |
| *Hoya griffithii* | 25 | 25 |  |  |
| *Hoya kerrii* | 25 | 25 |  |  |
| *Hoya lacunosa* | 25 | 25 |  |  |
| *Hoya lanceolata subsp. bella* | 29 | 21 |  |  |
| *Hoya liangii* | 25 | 25 |  |  |
| *Hoya longifolia* | 25 | 25 |  |  |
| *Hoya meliflua subsp. fraterna* | 25 | 25 |  |  |
| *Hoya ovalifolia* | 25 | 25 |  |  |
| *Hoya pandurata* | 25 | 25 |  |  |
| *Hoya pottsii* | 26 | 24 |  |  |
| *Hoya pubicalyx* | 26 | 24 |  |  |
| *Hoya radicalis* | 25 | 25 |  |  |
| *Hoya rigida* | 24 | 26 |  |  |
| *Hoya silvatica* | 26 | 24 |  |  |
| *Hoya* sp. 2 ZCF6006 | 25 | 25 |  |  |
| *Hoya* sp. 4 ZCF6004 | 27 | 23 |  |  |
| *Hoya* sp. 8 ZCF6076 | 25 | 25 |  |  |
| *Hoya* sp. 11 ZCF6107 | 26 | 24 |  |  |
| *Hoya thomsonii* | 25 | 25 |  |  |
| *Hoya volubilis* | 24 | 21 | 3 | 2 |
